# Supplementary material for: The genome sequence of the Fern moth, Horisme tersata (Denis & Schiffermüller) , 1775 (Lepidoptera: Geometridae)
Source: Wellcome Open Res. 2025 Oct 10;10:565. [Version 1] doi: 10.12688/wellcomeopenres.24980.1 (PMC12699213; doi:10.12688/wellcomeopenres.24980.1)
Supplement: Peer review history

# Reviewer report: Reviewer response for version 1

**DOI:** 10.21956/wellcomeopenres.27515.r140355

**Published:** 2025-12-11

**Authors:** Niclas Backström

**Affiliations:** 1 Uppsala University, Uppsala, Sweden

The report presents a high quality genome assembly of a male fern moth (Horisme tersata) and the procedures for data generation, assembly, quality assessment and report writing follow the standards of DToL. There are no major concerns regarding the methods or the assembly per se, I only have a couple of minor questions / suggestions (see below).

 Is this species only found in Britain - if not, maybe its worth mentioning briefly what the entire distribution range is?

 Second paragraph under Hi-C library preparation and sequencing. The second sentence (starting with "Normalised libraries were....") seems to be missing some information?

 Is the chromosome number expected based on karyotype information? 21 chromosomes is a small number compared to e.g. the ancestral lepidopteran karyotype, but maybe this is expected. A brioef comment on this would be informative.

 What was the rationale behind selecting a male individual for sequencing? To get similar coverage between the Z and the autosomes?

Are sufficient details of methods and materials provided to allow replication by others?

Yes

Is the rationale for creating the dataset(s) clearly described?

Partly

Are the datasets clearly presented in a useable and accessible format?

Yes

Are the protocols appropriate and is the work technically sound?

Yes

Reviewer Expertise:

Evolutionary genetics

I confirm that I have read this submission and believe that I have an appropriate level of expertise to confirm that it is of an acceptable scientific standard.

# Reviewer report: Reviewer response for version 1

**DOI:** 10.21956/wellcomeopenres.27515.r135921

**Published:** 2025-10-27

**Authors:** Marko Mutanen

**Affiliations:** 1 University of Oulu, Oulu, Northern Ostrobothnia, Finland

This report introduces a genome sequence for a Geometrid moth Horisme tersata. The genome is read from a male specimen, and while this is fine, a female specimen should be preferred to with species like this where obtaining a female is rather straightforward. 

 The species introduction is a bit brief and nation (UK) focussed. It shortly introduces the species' appearance and makes a short account of the species' habitats and food plant. The food plant information is not accurate in the wider perspective as the species is common in some areas where all Clematis spp. are absent. It has actually been documented to feed e.g. on Anemone nemorosa. Like usually (but in my opinion not desirably), the species' systematics or phylogenetics affinities are not touched, although its close relatedness to H. radicaria is discussed.

 The genome is clearly of good quality, as indicated e.g. by the high recovery rate of USCO loci. I have no reservations on that part.

Are sufficient details of methods and materials provided to allow replication by others?

Yes

Is the rationale for creating the dataset(s) clearly described?

Partly

Are the datasets clearly presented in a useable and accessible format?

Yes

Are the protocols appropriate and is the work technically sound?

Yes

Reviewer Expertise:

Lepidoptera systematics and taxonomy, DNA barcoding, species delimitation, species identification, dark taxa, phylogenetics

I confirm that I have read this submission and believe that I have an appropriate level of expertise to confirm that it is of an acceptable scientific standard.
